# List randomization for eliciting HIV status and sexual behaviors in rural KwaZulu-Natal, South Africa: a randomized experiment using known true values for validation

**DOI:** 10.1186/s12874-018-0507-9

**Published:** 2018-05-25

**Authors:** Noah Haber, Guy Harling, Jessica Cohen, Tinofa Mutevedzi, Frank Tanser, Dickman Gareta, Kobus Herbst, Deenan Pillay, Till Bärnighausen, Günther Fink

**Affiliations:** 1 Department of Global Health and Population, Harvard T.H. Chan School of Public Health, Boston, USA; 2 Carolina Population Center, University of North Carolina, Chapel Hill, USA; 3 Africa Health Research Institute, KwaZulu-Natal, South Africa; 4 Department of Epidemiology, Harvard T.H. Chan School of Public Health, Boston, USA; 5 Institute for Global Health, University College London, London, UK; 6 School of Nursing and Public Health, University of KwaZulu-Natal, Durban, South Africa; 7 Centre for the AIDS Programme of Research in South Africa—CAPRISA, University of KwaZulu-Natal, Congella, South Africa; 8 Division of Infection and Immunity, University College London, London, UK; 9 Institute of Public Health, University of Heidelberg, Heidelberg, Germany; 10 Research Department of Infection and Population Health, University College London, London, UK; 11 Swiss Tropical and Public Health Institute, University of Basel, Basel, Switzerland

**Keywords:** List randomization, Item count, Social desirability bias, HIV/AIDS, Sexual behaviors, Survey methods, South Africa

## Abstract

**Background:**

List randomization (LR), a survey method intended to mitigate biases related to sensitive true/false questions, has received recent attention from researchers. However, tests of its validity are limited, with no study comparing LR-elicited results with individually known truths. We conducted a test of LR for HIV-related responses in a high HIV prevalence setting in KwaZulu-Natal. By using researcher-known HIV serostatus and HIV test refusal data, we were able to assess how LR and direct questionnaires perform against individual known truth.

**Methods:**

Participants were recruited from the participation list from the 2016 round of the Africa Health Research Institute demographic surveillance system, oversampling individuals who were HIV positive. Participants were randomized to two study arms. In Arm A, participants were presented five true/false statements, one of which was the sensitive item, the others non-sensitive. Participants were then asked how many of the five statements they believed were true. In Arm B, participants were asked about each statement individually. LR estimates used data from both arms, while direct estimates were generated from Arm B alone. We compared elicited responses to HIV testing and serostatus data collected through the demographic surveillance system.

**Results:**

We enrolled 483 participants, 262 (54%) were randomly assigned to Arm A, and 221 (46%) to Arm B. LR estimated 56% (95% CI: 40 to 72%) of the population to be HIV-negative, compared to 47% (95% CI: 39 to 54%) using direct estimates; the population-estimate of the true value was 32% (95% CI: 28 to 36%). LR estimates yielded HIV test refusal percentages of 55% (95% CI: 37 to 73%) compared to 13% (95% CI: 8 to 17%) by direct estimation, and 15% (95% CI: 12 to 18%) based on observed past behavior.

**Conclusions:**

In this context, LR performed poorly when compared to known truth, and did not improve estimates over direct questioning methods when comparing with known truth. These results may reflect difficulties in implementation or comprehension of the LR approach, which is inherently complex. Adjustments to delivery procedures may improve LR’s usefulness. Further investigation of the cognitive processes of participants in answering LR surveys is warranted.

**Electronic supplementary material:**

The online version of this article (10.1186/s12874-018-0507-9) contains supplementary material, which is available to authorized users.

## Background

Self-reported data, particularly for questions in stigmatized areas, are often subject to social desirability bias, inducing individuals to give answers to survey questions which are influenced by societal preferences [1, 2]. Social desirability and related biases are particularly relevant for sexual behaviors and HIV research [3–5]. To address this issue, survey methodologies have been designed to make participants feel more comfortable giving truthful answers, generally by modifying the answer format in a way that makes it hard or impossible for the interviewer to know the participant's individual answer to the sensitive question. One method that has been receiving recent attention is list randomization (LR), also referred to as item or unmatched count technique. LR embeds responses to sensitive questions into a longer list including non-sensitive questions, and then asks respondents to report the total number of correct statements in a given list. Because subjects only reveal a count of several questions and not answers to specific questions, interviewers and researchers are unable to distinguish to which individual questions the participant answered true/false, and social desirability bias should be minimized.

To infer average responses to sensitive questions, LR employs randomization of subjects into two arms to achieve the masking of the responses. In the first arm, participants are given a block of true/false questions, one of which is the sensitive item of interest (e.g. “Did you use a condom during your last sexual encounter?”). In the other arm, participants are typically given the same list, but without the sensitive question. Participants are then asked to indicate the number of questions which are true, without revealing which specific items are actually true. As long as subjects in each arm have the same characteristics – which should be achieved by randomly assigning arms – the difference in the mean counts across the two lists should correspond to the percentage of individuals for whom the sensitive question is actually true. In addition to population percentages, efficient multivariate regression and related statistical models are possible using these data with additional assumptions [6–9].

There is limited evidence of whether LR improves self-reported elicitation of sensitive behaviors with validation against known truths in public health, with mixed results [10–13]. In HIV, A Arentoft, et al. [13] found that LR yielded lower estimates of negatively stigmatized behaviors in HIV+ patients compared with directly asked questions, counter to their initial hypothesis. While this study used a proxy for known truth by directly observing adherence data after the survey was completed, they did not directly observe the retrospective adherence behavior asked about in the survey. Evidence in low and middle-income countries is similarly mixed and limited, with only two published studies identified using LR [14, 15], only one of which, TN Randrianantoandro, et al. [14], compared results with direct questioning. The item count process may be cognitively and logistically difficult in comparison with direct questionnaires, as LR introduces additional opportunities for error due to lack of education, language, and cultural appropriateness of both the sensitive and non-sensitive questions [16].

No studies published to date validate LR against individual known actual statuses of its participants. Only one study we have identified, B Rosenfeld, et al. [17], attempted to validate the method against known population data using voting results. This study asked participants to reveal how they voted, and used aggregated elections results as the known truth comparison, finding that list randomization improved estimates over direct questionnaires. However, while this study had known truths among the geographic area represented by its participants, it did not know the true voting status of its individual participants.

This study takes advantage of pre-existing individual-level data on HIV test participation and serostatus to, for the first time, validate LR estimates for two outcomes likely to face considerable desirability bias. We further compare the performance of LR to that of standard questionnaire responses. We conducted this study within the Africa Health Research Institute (AHRI) demographic surveillance site (DSS), a resource-poor, high HIV prevalence area in rural KwaZulu-Natal.

## Methods

### Setting and population

The study was conducted within the borders of the AHRI DSS in rural KwaZulu-Natal. The DSS covers an open cohort of over 100,000 people in a 438km^2^ region near Mtubatuba, South Africa. The primary ethnicity/language of the region is Zulu. The region is mostly rural and semi-urban and among the poorest in South Africa, with an estimated HIV prevalence of 29% [18, 19]. Participants were recruited from those participating in the annual DSS individual surveillance survey data collection round, consisting of all residents aged 15 and over and living within the borders of the DSS. In addition to demographic, socioeconomic, and health related questionnaires, the AHRI DSS performs annual surveillance HIV tests, the results of which were not disclosed to participants in 2016. Individuals in AHRI DSS are also linked to the Department of Health HIV treatment clinics in the area. Individuals who have records related to HIV treatment through this system have voluntarily entered the HIV related clinical setting and have received HIV related services. We therefore assume that these individuals know that they are HIV positive.

### Sample generation protocol

The study sample generation and recruitment procedure was designed to test the validity of the list randomization using known truth, rather than to create generalizable population estimates, in the ACDIS surveillance population. Participants are selected into this study through three levels. The first level contains the subset of 30,828 adult (18+) individuals who had participated in the ongoing 2016 AHRI DSS surveillance round from January 19, 2016 to September 1, 2016. The second level selects 8000 random individuals from that dataset, oversampling individuals with known HIV status and testing behavior from the HIV testing module in the 2016 round of ACDIS, resulting in our target sample. The final selection is based on a geographically diverse selection of 500 individuals across the ACDIS geographic area.

The sampling procedure was designed in conjunction with AHRI field work teams to balance maximizing the sample size of individuals for whom truth is recorded and linkable, diversity of the population from which the sample is drawn, and efficient recruitment of individuals to our study. The size of both the target sample (8000) and the study sample (500) were determined through a combination of simulation of expected responses and experience from the AHRI fieldwork teams to most efficiently allocate fieldwork resources with respect to study goals. While it would have been theoretically feasible to start with a stratified list of 500 randomly selected individuals, field experience suggested that this would result in a resource intensive procedure, as it would require contacting individuals, scheduling visits, and inefficient travel for field workers. Instead, the protocol outlined below allowed field workers to maximize the number of visits per day, ensure geographic diversity, and maintaining internal validity through arm randomization, at the cost of generalizability.

### Target sample generation

We used a stratified-random selection scheme to generate the 8000-person target sample from those 30,828 individuals meeting our inclusion criteria. The individuals in the 2016 ACDIS dataset were divided into four strata, as below:

1. Those who tested HIV positive in surveillance and were linked to HIV treatment system (i.e. those who are positive and know their HIV status)
2. Those who tested HIV positive in surveillance and were NOT linked to HIV treatment system (i.e. those who are positive and may or may not know their HIV status)
3. Those who tested HIV negative in surveillance
4. Those who did not test (i.e. those who refused the surveillance HIV test).

2000 individuals were randomly selected from each of the above four categories of individuals, yielding 8000 individuals in the target sample, 25% from each of the above categories.

### Study sample generation

The fieldwork team was given a list of the 8000 individuals in the target sample with their names, field identifier, sex, and approximate locations, but no other information. A fieldwork coordinator was instructed and trained to manage the collection of 500 surveys from across the ACDIS region. The coordinator assigned each fieldworker a daily list of assigned individuals to approach, generally by geographic sub-region, with fieldworkers approaching a different set of individuals each day. Daily assignments were designed and adjusted to both maximize geographic diversity of the sample within the ACDIS borders when the pre-determined stopping rule of 500 surveys collected was reached. Fieldworkers were instructed to attempt to visit a given individual only once, skipping individuals if they were not available and/or refused. Individuals were entered into the study sample if the individual was available, consent was given at the time of the household visit, and their survey data were successfully recorded and transferred to the secure data server.

### Randomization to experiment arms

The 8000 people in the target sample were randomized to one of two arms before being approached for recruitment into the study: Arm A (60%) and Arm B (40%) as described below. Randomization was stratified by the above four categories for the target sample generation. The arm to which participants had been randomized was not known to field workers until consent was obtained and electronic data capture had begun.

### Arm a

**Fig. 1:**
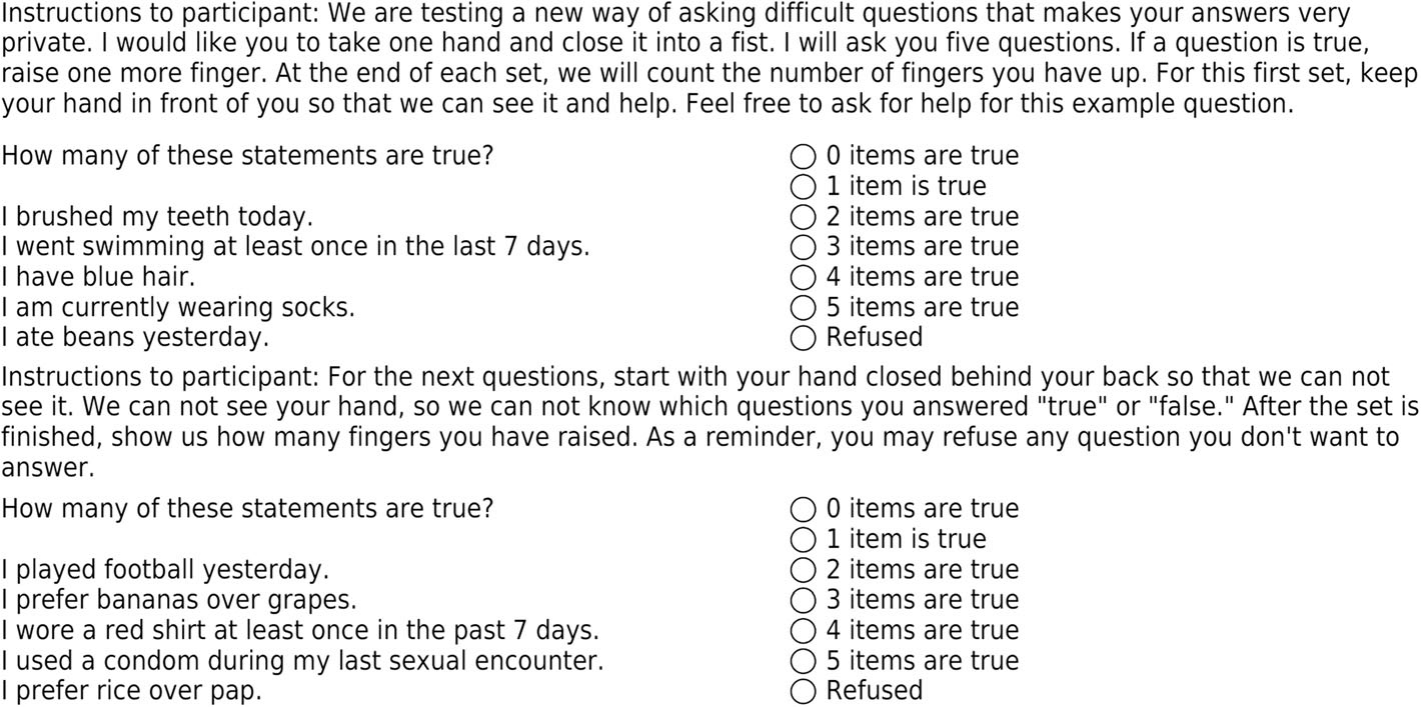
Sample list randomization question. This shows an example list randomization list block question

In this arm, participants were given five blocks, each block with five true/false subquestions. One subquestion in each block was the sensitive item, and the remaining four were non-sensitive questions. The first block, corresponding to the marginally sensitive question “Did you brush your teeth today” was used as a tutorial question. As suggested in T Tsuchiya, et al. [20] and T Nepusz, et al. [21], participants were asked to count on their fingers behind their backs as individual items within each block were asked. When the list of five sub-questions was finished, participants were asked to reveal their fingers to the surveyor. Five total blocks were given, one for each sensitive question. Figure 1 shows a sample list block question, including the training question and instructions.

The non-sensitive questions were selected and designed in conjunction with community representatives to be culturally relevant, easy to understand and answer, and be unlikely to be correlated with the sensitive question of interest. Independence of non-sensitive and sensitive questions allows both for fewer statistical modelling assumptions when estimating LR-based regressions and is a required condition for the design effects estimator, as discussed below. Using non-sensitive items which are topically irrelevant to the sensitive question improves the plausibility of this independence assumption [7].

It is plausible that having sensitive questions which are topically different than the non-sensitive questions may induce additional cognitive effects by calling attention to the sensitive question. To test this hypothesis, we randomize the position in each block in which the sensitive question appears (i.e. first through fifth item within each block). If the degree to which sensitive questions stand out changes responses, we might similarly expect that the ordering of those questions would impact responses, assuming that the ordering also impacts the degree to which sensitive questions stand out.

One particularly challenging aspect in the design of LR surveys are ceiling/floor effects which occur when participants’ count in a given block approaches extremes (in this case 0 or 5 true/affirmative). In cases where subjects give all affirmative or all negative answers to non-sensitive questions, the actual answers to the sensitive question can be easily inferred. Given this, non-sensitive items should be chosen such that most subjects have one to three (out of four) affirmative answers across the non-sensitive items. Using assumed probabilities of affirmative answers for each question generated by discussion with community representatives, simulations were performed with random assignment of non-sensitive questions to blocks, and the assignment that had the lowest simulated probability of producing extreme responses (0 or 5) was selected. Estimated probabilities used for this simulation are shown in Additional file 1 alongside the full list of questions and associated blocks.

### Arm B

Arm B serves two main purposes: estimating of the true/false counts for the non-sensitive questions, and estimating the true/false counts for the sensitive question. Arm B asks all questions, both sensitive and non-sensitive questions, directly. Each of the non-sensitive questions in Arm B is a component in one of the blocks from Arm A, allowing counts to be generated for the non-sensitive questions. Asking the sensitive questions directly allows comparison of the LR-estimated percentages compared to standard, directly-asked questionnaires. These questions are asked after the non-sensitive questions to ensure that they do not influence the non-sensitive answers.

This format differs from many other list randomization studies, which typically have a secondary arm identical to the first but without the sensitive question. The standard design helps ensure that if there are cognitive biases due to the counting procedure, biases would be roughly equal in both arms. However, unlike other list experiments, this study is interested in the exact percentages of non-sensitive items in this population to inform the construction of future list experiments in this population, designing questions which avoid ceiling and floor effects. Further, this study design allows for the potential use of alternative estimators, such as that proposed in D Corstange [22], which take advantage of individually asked questions to potentially improve efficiency of multivariate regression models in list experiments.

### Sensitive items of interest

The five sensitive questions of interest are listed below. The first question below is used as a training question, and as such does not contain truly sensitive information.

- I brushed my teeth today. (+)
- I used a condom during my last sexual encounter. (+)
- I am HIV negative. (+)
- I have had anal sex within the last 12 months. (−)
- I refused the AHRI DSS HIV test this year. (−)

We expected a positive social desirability bias for the first three questions, and a negative social desirability bias associated with the latter two questions, as indicated by the +/− signs above. We define an improvement in inference in this paper as when the LR estimate yields estimates for which at least one of the following is true: LR estimates lower percentages estimated for items with an expected positive social desirability bias, LR estimates higher percentages estimated for items with an expected negative social desirability bias, or LR estimates are closer to the actual value when known, as compared with direct questionnaire estimates.

### Survey procedures

Fieldworkers approached target individuals at their homes, proceeding with the survey only if signed consent was obtained. Participants signed consent via electronic tablet, and were given a physical copy of the consent form and survey information. The survey and the electronic signature consent were administered by electronic tablets using REDCap™ software for data capture. All instructions, questions, and consent were given in Zulu, as translated by local native Zulu speakers in the community engagement team at AHRI. Fieldwork was considered complete when field workers reported 500 surveys completed. Both versions of the survey keep track of time to response in order to assess cost of implementation in future surveys.

### Human subjects and IRB approval

The protocol for this survey was approved by the University of KwaZulu-Natal BioMedical Research Ethics Committee (BF291/16) and by the Harvard University Institutional Review Board (IRB16–0864).

### Statistical analysis

The main outcome of interest is the estimated prevalence for key HIV-related outcomes. We use two estimators of the prevalence of the sensitive items in our surveys: list randomization and direct questionnaire. To estimate the prevalence of a sensitive item using list randomization, we use a variation of the difference in means approach [7], which utilizes information from both Arm A and Arm B. For a given block of questions, we have two variations in how the block was asked. For Arm A, which contains the sensitive question, we simply take the mean of the counts of affirmative answers for each block. For Arm B, we take the sum of affirmative responses for each person corresponding to the four non-sensitive questions in the block, and take the mean of these counts. The direct questionnaire estimate uses only the direct questions about the sensitive item from Arm B alone.

The main multivariate regression is performed using the linear regression methodology from G Blair, et al. [23] to estimate correlations between sensitive question answers as elicited by the LR method and both known truth and demographic information. This will help assess the degree to which answers are at least correlated with known truths by including actual HIV status/refusal as a covariate. In the case of HIV status, actual status variables cannot be included in the population with HIV status known to the participant, as all of these individuals are HIV+. The linear model is chosen for computational robustness. While alternative models, such as the K Imai (2011) [8] MLE estimator, may provide improved statistical efficiency, these models may introduce computational difficulties, and are treated as tertiary experimental methods in this analysis.

Finally, we test for the possible presence of design effects using G Blair, et al. [7]‘s proposed design effects estimator, which attempts to detect the presence of individuals giving different answers to the sensitive question due to the design of and/or results of the non-sensitive questions. Ceiling and floor effects, for example, are scenarios in which extreme counts of responses (i.e. all true/affirmative or all false/negative) could reveal the respondents’ response to the sensitive question. G Blair, et al. [7]‘s design effects test attempts to detect the existence of these effects by exploiting differences in the expected probability of positive/negative responses at each count level of non-sensitive questions. Higher percentages of expected negative probabilities at different count levels may indicate design effects, especially those related to non-sensitive question counts. However, this test would not necessarily detect other biases caused by the design of the survey and is of limited statistical power given our sample size.

Actual known status percentages for HIV status and test refusal are estimated for all subpopulations. For comparability with the LR estimates, these percentages are treated as estimates of the percentages from the underlying population and data generation process with associated standard errors/confidence intervals. We estimate the full sample percentage of HIV positive individuals by assuming that those in our sample who refused the HIV surveillance test had the same percentage HIV positive/negative as the ACDIS general population in 2016. Applying this percentage to our sampled “refuse” population yields an estimated percentage positive/negative for our full sample. We use the width of the confidence intervals from the non-refused sample population as a conservative (i.e. too wide) estimate of the confidence intervals around this population, under the conservative assumption that the application of the assumed general population adds no precision to our population estimate.

Point estimates and estimated standard errors for all statistics are taken only within the context of this study with respect to its sampling structure, and therefore estimates were not adjusted for sampling weights and stratification. Unless otherwise noted, all results are from the study sample.

All methods presented here, unless otherwise noted, are implemented in R, with all LR-specific calculations using the “list” package [23].

## Results

Data collection stopped when field workers reported having completed questionnaire for 500 individuals across the AHRI DSS, out of the list of 8000 possible target individuals, after which data collection was considered completed. After data collection completion, 483 completed survey records were available and extracted from the REDCap™ server, with an additional 13 individuals recorded as refusing consent when offered. The 17-person (3.4%) discrepancy between the 500 individuals reported by field workers vs. the 483 completed records extracted is likely due to a combination of data transfer from the tablets to the server and/or errors in reporting of survey completion.

Table 1 shows summary statistics for study participants. Of those who participated and were recorded, 262 (54%) received Arm A, and the remaining 221 (46%) received Arm B. Demographics were similar across both randomized arms, with a mean age of 40, 36% males, and 8.7 years of education, and broadly similar to the target sample. Those in Arm A took a total of 302.1 s to complete the survey, including instructions, as compared with 42.2 s to complete direct questioning of the four sensitive items of interest in Arm B (not including instructions). Additional details of the times are shown in Additional file 1. 22% of the total sample were verified HIV negative by the surveillance HIV test, with 26% of participants were HIV negative among those who did not refuse the HIV test. We assume that the 71 individuals who refused the 2016 round of surveillance had the same percentage HIV negative/positive as the general ACDIS population (31%), yielding an adjusted full sample HIV negative estimate of 32.1%.

**Table 1:** Descriptive statistics

	Study sample				Target sample
	All	Arm A	Arm B	Difference A vs. B	All
	mean (SD)	mean (SD)	mean (SD)	difference (*p*-value)	mean (SD)
Age	40 (16)	40 (17)	40 (15)	0 (0.83)	39 (15)
Male	0.36 (0.48)	0.34 (0.48)	0.38 (0.49)	− 0.04 (0.41)	0.46 (.50)
Education (years)	8.7 (4.0)	8.5 (4.2)	8.9 (3.8)	−0.4 (0.34)	8.9 (4.0)
Employed	0.28 (0.45)	0.27 (0.45)	0.29 (0.46)	−0.02 (0.63)	0.34 (0.47)
Tested HIV positive^a^	0.63 (0.48)	0.66 (0.48)	0.61 (0.49)	0.05 (0.26)	0.50 (0.50)
Tested HIV negative^a^	0.22 (0.41)	0.20 (0.40)	0.24 (0.43)	−0.04 (0.32)	0.25 (0.43)
HIV test refused^a^	0.15 (0.35)	0.14 (0.35)	0.15 (0.36)	−0.01 (0.70)	0.25 (0.43)
Linked to HIV care	0.34 (0.47)	0.31 (0.46)	0.37 (0.48)	−0.06 (0.15)	0.25 (0.43)
n	483	262	221		8000

SD = Standard Deviation. *P*-value for differences were estimated with a two-tailed t-test. ^a^Testing HIV positive, negative, and refusing the test are mutually exclusive categories

**Fig. 2:**
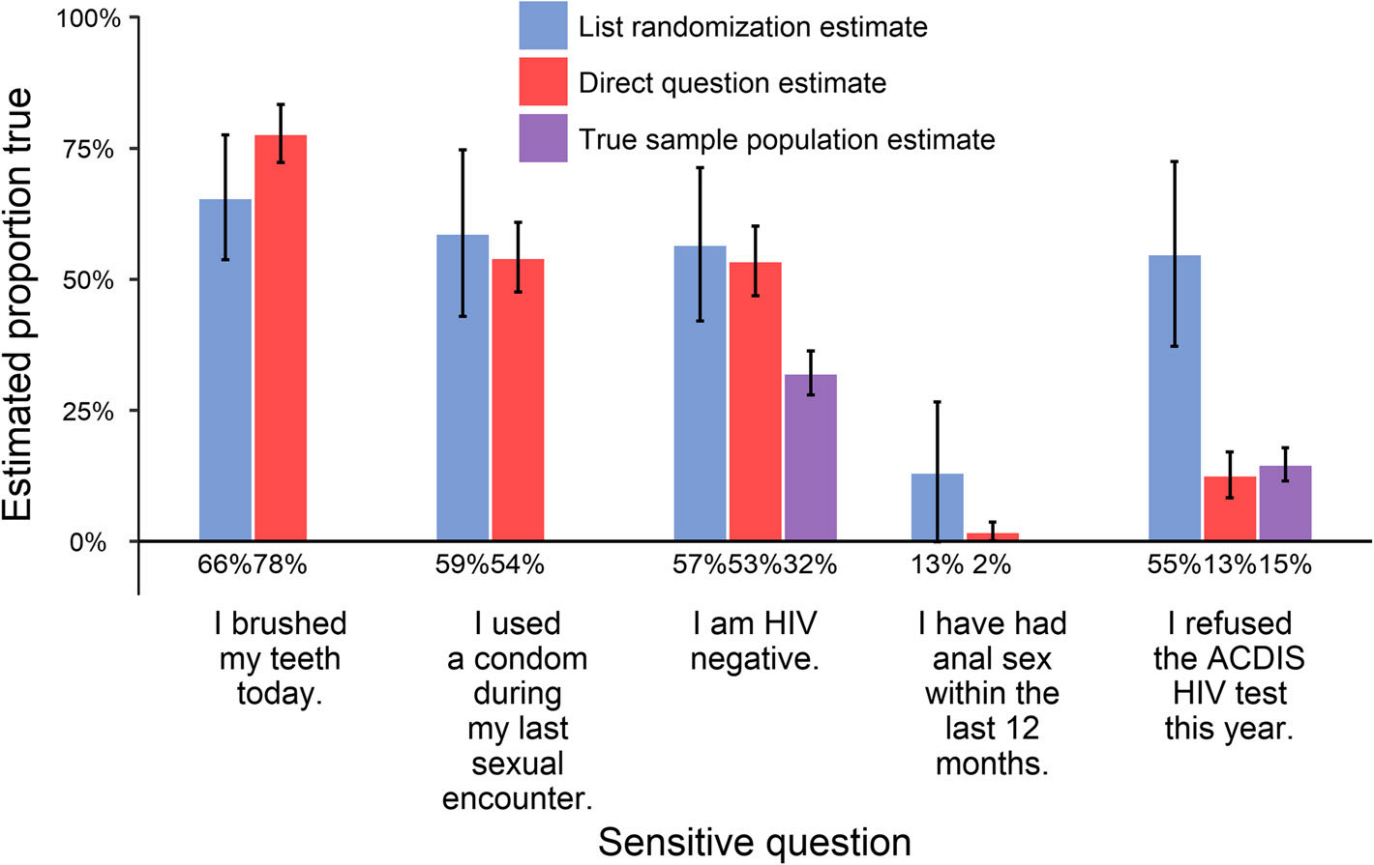
Estimated percentage true for all sensitive questions. Bars show the estimated percentage of the population answering “true” (affirmative) to the sensitive question, using the estimation method represented by the color of the bar. 95% confidence interval in brackets

As shown in Fig. 2, LR estimates did not yield an improvement in inference on any of the five sensitive questions. Each column in Fig. 2 represents the estimated percentage responding in the affirmative for the sample population for each of the five sensitive questions, using LR, direct estimation, or actual values where available. Magnitudes of LR estimates were similar to the direct questionnaire estimates for four out of the five sensitive questions, and were further from the truth for the remaining question. Standard errors and 95% confidence intervals were much larger across the board than the direct questionnaire, although CIs overlap between direct and LR estimated probabilities in all cases except the last. Neither of the questions for which truth is known yielded improved estimates. For the question regarding being HIV negative, LR estimated 56% (95% CI: 40 to 72%) of the population to be HIV-negative, compared to 47% (95% CI: 39 to 54%) using direct estimates; the population-estimate of the true value was 26% (95% CI: 22 to 30%). For the final question regarding HIV test refusal, however, the estimated percentage of affirmative answers were qualitatively worse in the LR estimate as compared to the direct questionnaire estimate. The LR version estimated 55% (95% CI: 37 to 73%) of individuals refused their last AHRI DSS HIV test, as compared with 13% (95% CI: 8 to 17%) in the direct group.

**Fig. 3:**
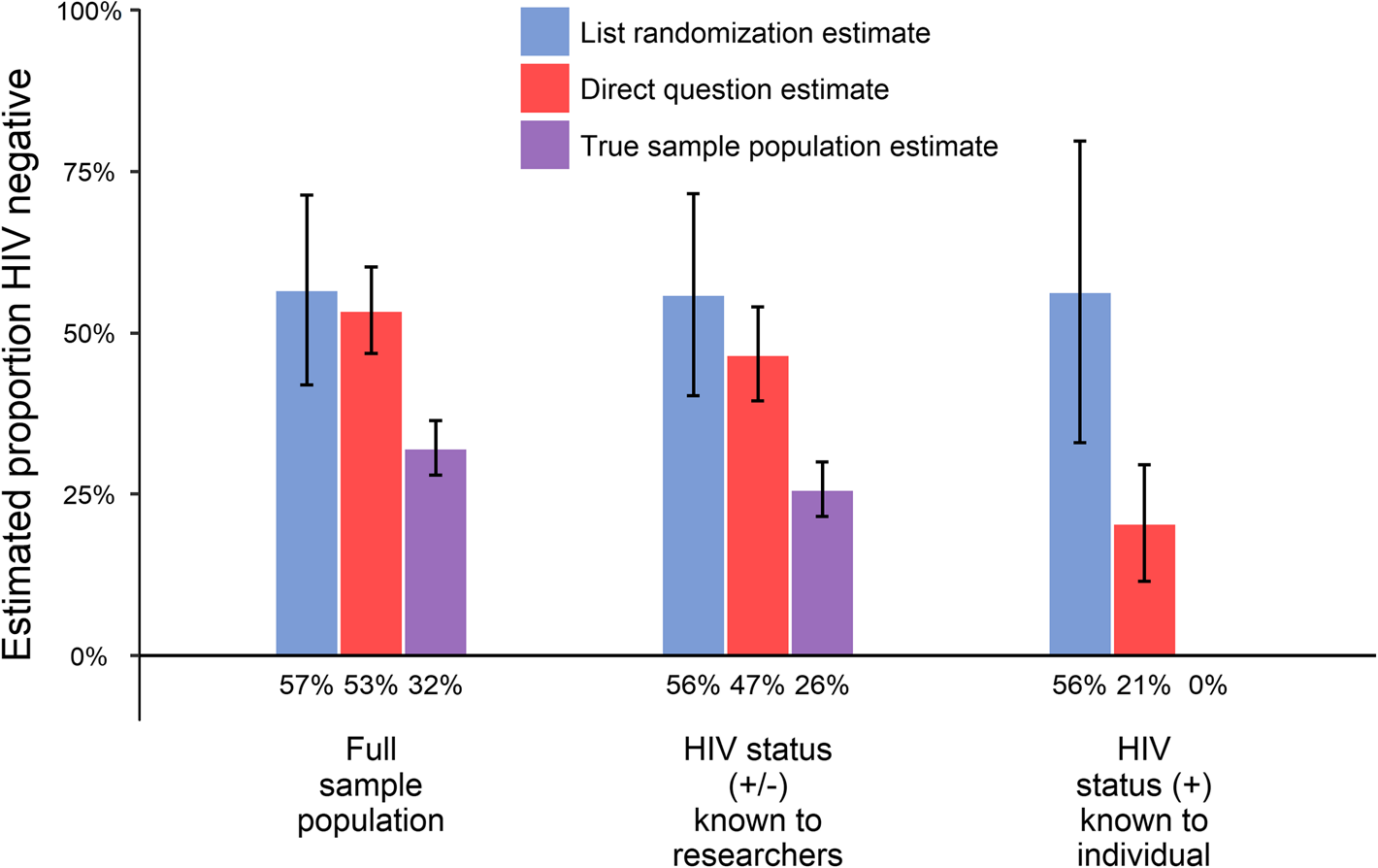
Estimated percentage affirmative for HIV status (“I am HIV negative”). Bars show the estimated percentage of the population answering “true” (affirmative) to the sensitive question, using the estimation method represented by the color of the bar, where the purple bar represents the true percentage. 95% confidence interval in brackets. Sub-populations are shown below each chart

Figure 3 compares the list randomization estimate and the direct questionnaire estimate to the actual known prevalence for HIV status, with each column showing the estimated proportion affirmative for the given question, population, and method. Both the list randomization and the direct questionnaires yielded overestimates of the percentage of individuals who were HIV negative, as compared to the actual levels of being HIV negative as shown in purpose. In middle panel, we include only individuals who did not refuse the previous AHRI DSS HIV test. These are the individuals in the sample for whom we have known HIV status, although we note that because these test results are not disclosed to the participant, participants may not necessarily know their status. In the right-hand panel, we only include individuals who tested HIV positive and have been linked to the local HIV clinic system. We assume these individuals are aware of their status as they have received HIV-related services at local clinic. We note that the confidence intervals for neither the LR nor direct questionnaires contain the true value of 0, but the LR estimate of the percentage who were HIV negative was very high, with an estimate of 56% (95% CI: 33 to 80%).

**Fig. 4:**
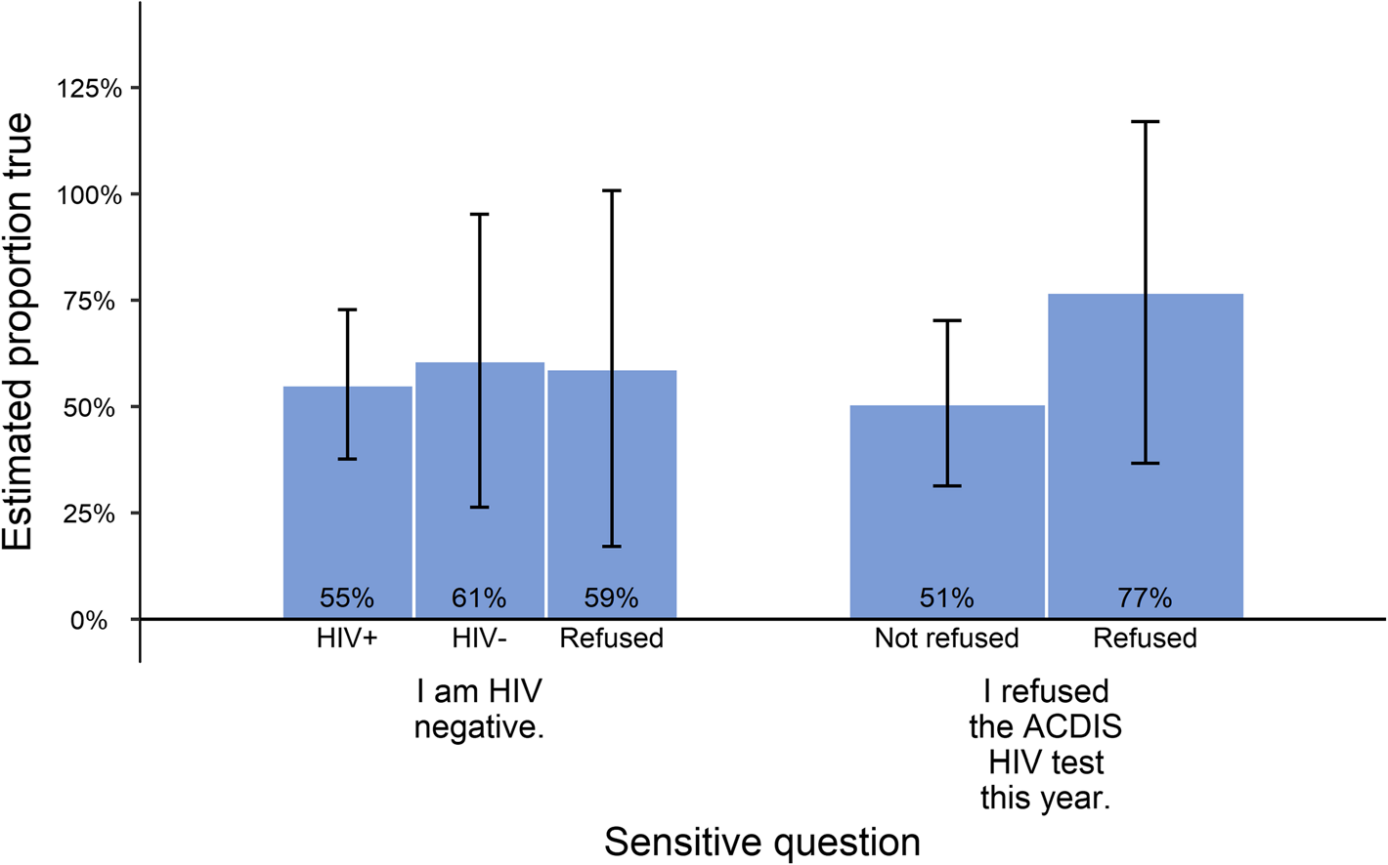
Estimated percentage affirmative by true status. Bars show the estimated percentage of the population answering “true” (affirmative) to the sensitive question. Subpopulations by HIV test status are shown by bar colors. 95% confidence interval in brackets. True status as shown in this chart is known to the researchers, but may not necessarily be known to the individuals

The percentage of individuals estimated to be HIV negative does not appear to be strongly correlated with actual HIV status, as shown in Fig. 4. Figure 4 is similar to the previous figures, but includes only the LR estimates for each of the relevant HIV results/refusal subpopulations. Furthermore, the LR method far overestimated the percentage who refused the AHRI DSS HIV test, as shown in Fig. 2. While the actual percentage was only 15% (95% CI: 12 to 18%), LR estimated 55% (95% CI: 37 to 73%).

Multivariate regression results with the list randomization data using the linear regression methodology [23] for the two questions with known truth are shown in Table 2. Elicited responses using the list randomization methodology are generally unresponsive to demographics, including gender, age, and education. Being HIV negative is non-significantly associated with elicited “yes” answers for “I am HIV negative,” as seen in columns (1–3). Years of education is positively associated with claiming being HIV negative in column (3), despite these individuals being HIV positive. Results are similarly non-significantly associated with actual HIV status using the theoretically more efficient MLE model from G Blair, et al. [7], noting that several specifications failed to converge. Having refused an HIV test is positively associated with elicited “yes” answers for the question regarding test refusal, but is only significant when demographics are controlled for in column (4). The results for the questions without known truth are shown in Additional file 1.

**Table 2:** Regression results for questions with known truth

Question / dependent variable	“I am HIV negative”			“I refused the AHRI DSS HIV test this year”
Subpopulation	Full population	HIV status (+/−) known to researchers	HIV (+) status known to individual	Full population
	(1)	(2)	(3)	(4)
Coefficient:				
Constant	0.67 (−0.57, 1.90)	0.82 (− 0.62, 2.27)	−1.36 (−4.73, 2.01)	−0.55 (−1.92, 0.83)
HIV negative	0.06 (−0.40, 0.52)	0.01 (− 0.45, 0.48)		0.43 (− 0.08, 0.93)
HIV test refused	0.08 (− 0.40, 0.55)			0.54** (0.03, 1.06)
Male	0.02 (−0.31, 0.34)	−0.05 (− 0.40, 0.30)	−0.16 (− 0.67, 0.36)	−0.08 (− 0.46, 0.31)
Age	− 0.01 (− 0.06, 0.03)	−0.02 (− 0.08, 0.03)	0.04 (− 0.13, 0.20)	0.05* (− 0.01, 0.10)
Age^2†^	0.02 (− 0.04, 0.07)	0.03 (− 0.03, 0.09)	−0.02 (− 0.21, 0.17)	−0.04 (− 0.10, 0.02)
Years of education	0.02 (− 0.04, 0.07)	0.02 (− 0.04, 0.08)	0.09** (0.01, 0.17)	−0.02 (− 0.08, 0.04)
Residual SE	0.84	0.83	0.75	0.98
Observations				
*Arm A*	242	209	77	245
*Arm B*	210	176	76	210
*Total*	452	385	153	455

Coefficients are shown as point estimate (95% confidence interval). * *p* < .10, ** *p* < .05. Correlation with non-sensitive item component not shown. Residual SE (standard error) is shown for model fit and ranges from 0 to 1, where a residual SE of 0 is a perfectly fit model. ^†^ coefficient multiplied by 100

Results are shown for the linear estimation model

**Fig. 5:**
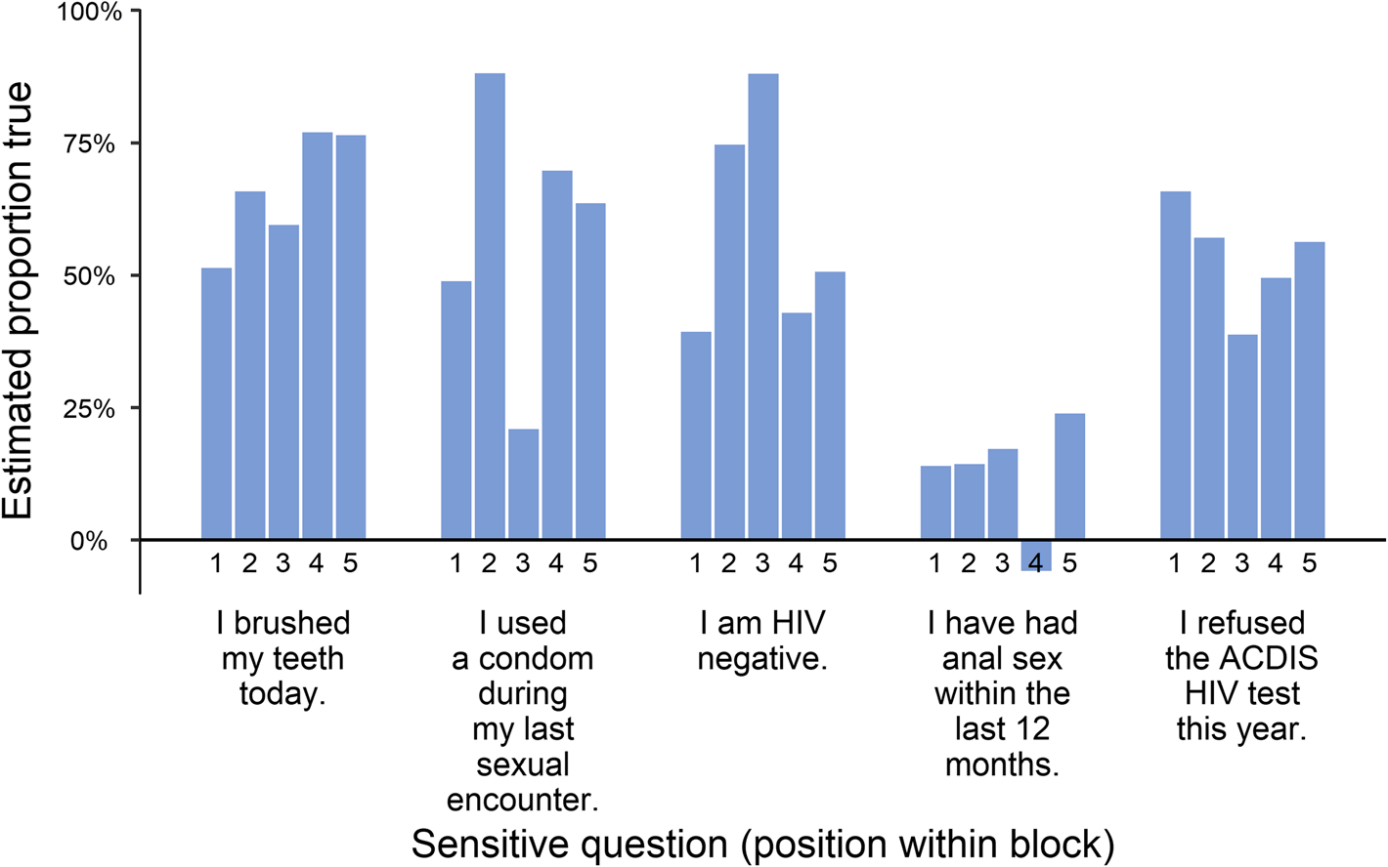
Percentage of participants answering 0–4 non-sensitive questions affirmative in Arm B. Each bar represented the percentage of sums which are equal to 0, where each sum is the sum of the four non-sensitive question “true” (affirmative) answers associated with a given sensitive question item block

While the question blocks were designed to minimize the probability of ceiling and floor effects, we found that the probability of extreme-valued sums was relatively high for questions pertaining to three of the blocks in Arm B. We show the percentage of participants answering 0/4 non-sensitive questions affirmative in Arm B in Fig. 5, noting that the opposite extreme (4/4) occurred in less than 1% of cases for all arms. For three of the blocks (brushing teeth, condom use, and HIV test refusal), more than 20% of respondents answered ‘no’ to all four non-sensitive questions. The only two question blocks for which there was both a high percentage of 0 sums and for which societal preference is positive are for brushing teeth and for condom use. We further do not observe statistically significant evidence for design effects using G Blair, et al. [7]‘s design effect test, with *p*-values for the test being 0.58, 0.16, 0.66, 0.10, and 0.59 for each block, respectively. Further, we do not observe any consistent pattern of the impact of the position of the sensitive question within blocks on LR estimates, as shown in Additional file 1.

## Discussion

We conducted a study in rural KwaZulu-Natal, South Africa comparing list randomization methods to direct estimates of prevalence for HIV-related outcomes, including a comparison to objectively measured individual values for these outcomes. In our study, LR responses neither corresponded strongly with known actual answers with regard to HIV status and HIV testing refusal, nor were closer to known actual answers than standard direct questionnaires. Our results agree with recent findings from A Arentoft, et al. [13], which similarly did not find that list randomization improved estimates over direct questioning. The failure of our implementation of LR to improve elicitation of sensitive information in surveys highlights the inherent complexity of this and other related methods, suggesting that researchers should be cautious when using them in the field.

Methods which add a randomized element to survey results share a trade-off between the degree to which answers are hidden, and therefore theoretically the degree of bias reduction, with loss of statistical power through some form of increasing measurement error [24]. In theory, by inducing semi-random error into participant responses, participants gain plausible deniability of their answers, and as such may feel less pressure to answer untruthfully. For LR, this is achieved by adding the binary responses of unrelated questions to participant responses and using a second set of individuals to serve as population controls, at least doubling the necessary population needed to achieve a given level of power. In our case, LR increased statistical noise, but did not reduce bias. We therefore conclude that the list randomization method as implemented was not useful in this setting.

The most substantial limitations to this survey were those of external validity, including limitations of generalizability to other populations, other question topics, and even other implementations of LR. Our study sample is drawn from a semi-urban, relatively poor, Zulu-speaking region of rural KwaZulu-Natal, limiting transportability to other settings. Further, we purposefully selected individuals with known actual answers to particular questions, with at least three levels of selection limiting generalizability of estimated survey answers even to the AHRI region: selection into the ACDIS household survey cohort, selection into the target sample based on ACDIS HIV tests, and finally selection into our study sample through being approached at home during working hours. It is likely that this cohort would be more likely to understand and accept the unusual line of questioning than other general populations, as these were individuals who had recently taken at least one test and survey about HIV as well as belonging to a region with a highly visible population research center. Questions were designed and selected based on the availability of known truth, and as such may not be applicable to other topics or questions. This study serves as a methods validation study only, and by design is inappropriate to make population level inference.

The specifics of the design and implementation of the list randomization questionnaire may help explain its poor performance in our experiment. While counts in the non-sensitive questions were unexpectedly low and could indicate floor effects, design effects alone cannot explain the discrepancy between known truths and list randomization-estimated percentages. Despite a priori estimation and simulation to avoid extreme counts, we noted unexpected percentages of respondents with summed blocks adding to extreme counts of affirmative responses present in Arm B. However, the expected direction of floor effect bias is not consistent with the expected direction of floor effect bias in the presence of social desirability for the question regarding HIV testing, and therefore cannot be the primary source of bias and/or error in this question. Similarly, the position of the sensitive questions within blocks did not appear to have a consistent pattern of impact on estimates, suggesting that drawing attention to the sensitive question was unlikely to be the primary driver of bias/error. We also note that using a list block (Arm A) and a direct arm (Arm B), as opposed to two list block arms, may have contributed to bias. Differences in the question formats may induce different biases in each arm, yielding a net bias when using the difference in means estimator. However, the data collected in Arm B will help inform future study designs in the ACDIS cohort so we are better able to avoid design effects in the future.

We speculate that the most substantial issue in our implementation was cognitive difficulty associated with the list block format. Participants in the LR arm were asked to keep a running tally on their fingers how many items were true, but also had to acknowledge that they heard and accounted for each sub question in the block. While this method was pre-tested and chosen as it required no additional materials, subjects’ ability to keep track of answers may have been insufficient. However, we did not find evidence that more education yielded improved responses, nor do we find that our sample is disproportionately educated. To make things easier for subjects, physical devices could have been used to reduce cognitive load. For example, participants could have been given two jars (for true and false answers), marbles, and a screen to block view from the interviewer, and instructions to drop a marble in the true and false jars as the sub questions are being asked, counting the number of marbles in each jar at the end of each block.

Finally, we hypothesize that in at least one case, the sensitive question itself may have contributed to the discrepancy in results. While patients were asked whether they refused the most recent AHRI DSS HIV test (i.e. “this year”), participants could have misunderstood this as meaning any other HIV test, including other rounds of tests. If this was the case, participants misunderstanding the question would be overly likely to believe their actual answer to be affirmative, while still having social pressure to answer “false.” The direct questionnaire in this case yielded answers that were unexpectedly close to the actual truth. While that could be the result of two sources of error in opposite directions cancelling each other out as above, it is alternatively plausible that there simply is not strong social desirability bias for this question. It is further plausible that recall may have had a role, as two of the questions used a recall period of 12 months [25].

While this implementation did not confirm the hypothesis that list randomization would improve validity of data, it does highlight the need for additional study. Both the LR and direct questionnaire methods failed to yield accurate estimates of HIV status. Given the relative limited instances of list randomization use worldwide to date, there exists a number of open questions regarding the feasibility and efficacy of variants of LR. These include the number and variance of the non-sensitive items [7, 16, 26] and situational appropriateness in a variety of settings. List randomization and other comparable methods have the potential to greatly improve inference, but these methods are inherently more complicated and more time consuming, yielding greater risk of error.

## Conclusions

Based on this study, it is unclear whether list randomization is a useful tool in settings and populations comparable to that of the AHRI DSS. Formal cognitive interviewing [27, 28] will be greatly beneficial in survey design going forward to help design, implement and test counting methods and question framing. Our results suggest that, until further study is performed to determine best practices in a wide variety of settings, researchers should be cautious to use this approach.

## Additional files

Additional file 1: Appendix tables and figures. PDF containing all appendix tables and figures. (PDF 1020 kb)

Additional file 2: Full dataset for analysis, formatted as a CSV file. This file contains the main collected data from the study, with all identifying and demographic information removed. Additional data and alternative formats may be available upon request, pending review of ethics agreements. (CSV 103 kb)

## Abbreviations

AHRI: Africa Health Research Institute
DSS: Demographic Surveillance Site
HIV: Human Immunodeficiency Virus
LR: List randomization
