# Supplementary material for: List randomization for eliciting HIV status and sexual behaviors in rural KwaZulu-Natal, South Africa: a randomized experiment using known true values for validation
Source: BMC Med Res Methodol. 2018 May 25;18:46. doi: 10.1186/s12874-018-0507-9 (PMC5968464; doi:10.1186/s12874-018-0507-9)
Supplement: Supplementary file 1 — Appendix tables and figures. PDF containing all appendix tables and figures. (PDF 1020 kb) [file 12874_2018_507_MOESM1_ESM.pdf]

Appendix 1: Questionnaire results summary

	a priori estimated % affirmative	Arm B survey estimated % affirmative	# (%) refusals
Block 1 (training question)			
I am currently wearing socks.	50%	15%	1 (0%)
I have blue hair.	0%	0%	1 (0%)
I went swimming at least once in the last 7 days.	20%	1%	1 (0%)
I ate beans yesterday.	20%	22%	1 (0%)
**I brushed my teeth today.**	70%	78%	0 (0%)
Non-sensitive question sum	0.90	0.38	1 (0%)
LR count sum	1.60	1.04	0 (0%)
Block 2			
I wore a red shirt at least once in the past 7 days.	40%	10%	1 (0%)
I prefer rice over pap.	40%	31%	0 (0%)
I played football yesterday.	20%	3%	1 (0%)
I prefer bananas over grapes.	50%	62%	0 (0%)
**I used a condom during my last sexual encounter.**	20%	54%	5 (2%)
Non-sensitive question sum	1.50	1.06	1 (0%)
LR count sum	1.70	1.65	1 (0%)
Block 3			
I am right-handed.	90%	92%	1 (0%)
I have a blue pair of shoes.	20%	6%	1 (0%)
It rained yesterday.	5%	45%	1 (0%)
I ate amasi yesterday.	20%	10%	1 (0%)
**I am HIV negative.**	30%	53%	6 (3%)
Sum non-sensitive questions	1.35	1.53	2 (1%)
List randomization count	1.65	2.10	4 (2%)
Block 4			
I have two arms.	99%	98%	0 (0%)
I prefer Maskandi over Isicathamiya music.	50%	73%	1 (0%)
I prefer aubergine over beet root.	50%	12%	0 (0%)
I wore a blue shirt at least once in the past 7 days.	20%	11%	0 (0%)
**I have had anal sex within the last 12 months.**	5%	2%	3 (1%)
Sum non-sensitive questions	2.24	1.94	1 (0%)
List randomization count	2.24	2.07	2 (1%)
Block 5			
I drank coffee today.	20%	9%	0 (0%)
My favorite color is blue.	60%	31%	1 (0%)
I prefer yam over sweet potato.	60%	53%	1 (0%)
Today is a Thursday.	20%	19%	1 (0%)
**I refused the AHRI DSS HIV test this year.**	70%	13%	0 (0%)
Sum non-sensitive questions	1.40	1.11	2 (1%)
List randomization count	2.10	1.66	1 (0%)

Appendix 2: Estimates by demographic group

Population	Estimates: LR	Estimates: Direct	Estimates: Known truth	Sample sizes: LR	Sample sizes: Direct	Sample sizes: Known truth
	estimate (CI)	*estimate (CI)*	*estimate (CI)*	*total n (list block arm + direct arm)*	total n	total n
Panel a: "I brushed my teeth today."						
All	66% (54:78%)	78% (72:83%)		482 (262+220)	221	
Female	71% (56:85%)	82% (75:88%)		308 (172+136)	137	
Male	57% (37:77%)	71% (62:81%)		174 (90+84)	84	
Age 18-24	49% (28:71%)	74% (59:88%)		88 (51+37)	38	
Age 25-39	60% (41:80%)	82% (74:90%)		174 (91+83)	83	
Age 40-64	78% (57:98%)	78% (69:87%)		185 (100+85)	85	
Age 65+	77% (22:131%)	67% (42:91%)		35 (20+15)	15	
Panel b: "I used a condom during my last sexual encounter."						
All	59% (43:75%)	54% (48:61%)		481 (261+220)	216	
Female	65% (46:85%)	51% (42:59%)		307 (171+136)	136	
Male	47% (20:75%)	60% (49:71%)		174 (90+84)	80	
Age 18-24	58% (20:95%)	69% (53:84%)		88 (51+37)	35	
Age 25-39	73% (45:101%)	63% (53:74%)		173 (90+83)	82	
Age 40-64	61% (37:86%)	48% (37:58%)		185 (100+85)	84	
Age 65+	-17% (-68:34%)	7% (-6:20%)		35 (20+15)	15	
Panel c: "I am HIV negative."						
All	57% (42:71%)	53% (47:60%)	26% (22:30%)	477 (258+219)	215	412
Female	55% (37:74%)	48% (39:56%)	26% (21:31%)	304 (169+135)	134	269
Male	58% (33:83%)	63% (52:74%)	25% (18:32%)	173 (89+84)	81	143
Age 18-24	58% (25:91%)	86% (75:98%)	47% (36:59%)	88 (51+37)	36	72
Age 25-39	45% (18:71%)	53% (42:64%)	17% (11:23%)	171 (88+83)	81	147
Age 40-64	66% (42:89%)	36% (26:47%)	17% (11:23%)	184 (100+84)	83	164
Age 65+	65% (14:117%)	73% (50:96%)	66% (48:83%)	34 (19+15)	15	29
Panel d: "I have had anal sex within the last 12 months."						
All	13% (0:27%)	2% (0:4%)		480 (260+220)	218	
Female	15% (-2:32%)	1% (-1:2%)		307 (170+137)	136	
Male	11% (-10:33%)	4% (0:8%)		173 (90+83)	82	
Age 18-24	20% (-15:54%)	0% (0:0%)		88 (51+37)	36	
Age 25-39	10% (-12:32%)	4% (0:8%)		173 (90+83)	82	
Age 40-64	11% (-10:32%)	1% (-1:3%)		185 (100+85)	85	
Age 65+	23% (-34:80%)	0% (0:0%)		34 (19+15)	15	
Panel e: "I refused the AHRI DSS HIV test this year."						
All	55% (37:72%)	13% (8:17%)	15% (12:18%)	480 (261+219)	221	483
Female	58% (36:79%)	15% (9:21%)	13% (9:17%)	307 (171+136)	137	309
Male	51% (20:81%)	10% (3:16%)	18% (12:24%)	173 (90+83)	84	174
Age 18-24	37% (-5:79%)	8% (-1:17%)	19% (11:27%)	88 (51+37)	38	89
Age 25-39	45% (15:75%)	16% (8:24%)	16% (10:21%)	172 (90+82)	83	174
Age 40-64	71% (42:99%)	9% (3:16%)	11% (7:16%)	185 (100+85)	85	185
Age 65+	75% (18:132%)	27% (4:50%)	17% (5:30%)	35 (20+15)	15	35

Appendix 3: Multivariate regression results for questions with no known truth

	I brushed my teeth today.			I used a condom during my last sexual encounter.			I have had anal sex within the last 12 months.		
Covariate:	(1)	(2)	(3)	(4)	(5)	(6)	(7)	(8)	(9)
Intercept	0.66**	0.67**	-0.24	0.59**	0.67**	0.65	0.13*	0.18**	1.43**
	(0.06)	(0.08)	(0.54)	(0.08)	(0.11)	(0.71)	(0.07)	(0.09)	(0.61)
HIV negative		0.00	0.08		-0.23	-0.07		-0.09	-0.19
		(0.15)	(0.16)		(0.19)	(0.22)		(0.18)	(0.20)
HIV unknown (refused)		-0.11	-0.11		-0.29	-0.16		-0.26	-0.21
		(0.17)	(0.18)		(0.23)	(0.25)		(0.18)	(0.20)
Male			-0.18			-0.13			0.00
			(0.13)			(0.17)			(0.14)
Age			0.04			0.01			-0.05
			(0.02)			(0.03)			(0.02)
Age^2			0.00			0.00			0.00**
			(0.00)			(0.00)			(0.00)
Years of education			0.01			0.00			-0.03
			(0.02)			(0.03)			(0.02)
coefficient (SE). * p<.10, ** p<.05. Correlation with non-sensitive item component not shown.									
Residual SE	0.68	0.68	0.66	0.90	0.90	0.88	0.76	0.76	0.76
Observations									
List block arm	262	262	246	261	261	245	260	260	245
Direct arm	220	220	211	220	220	211	220	220	211
Total	482	482	457	481	481	456	480	480	456

Appendix 4: Time to completion of each survey section

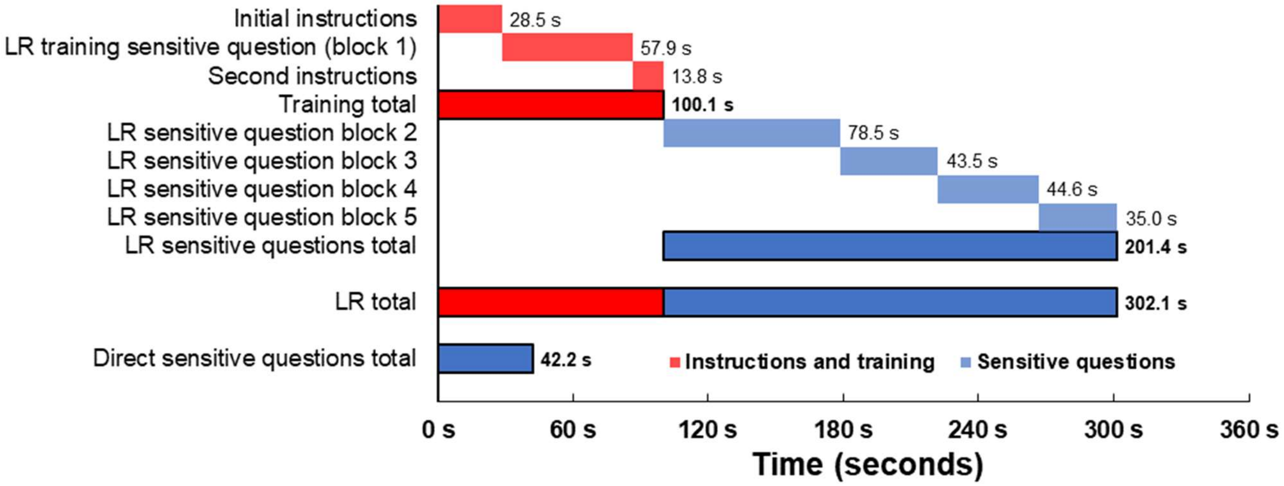

Appendix 5: LR estimate by position of sensitive questions within block

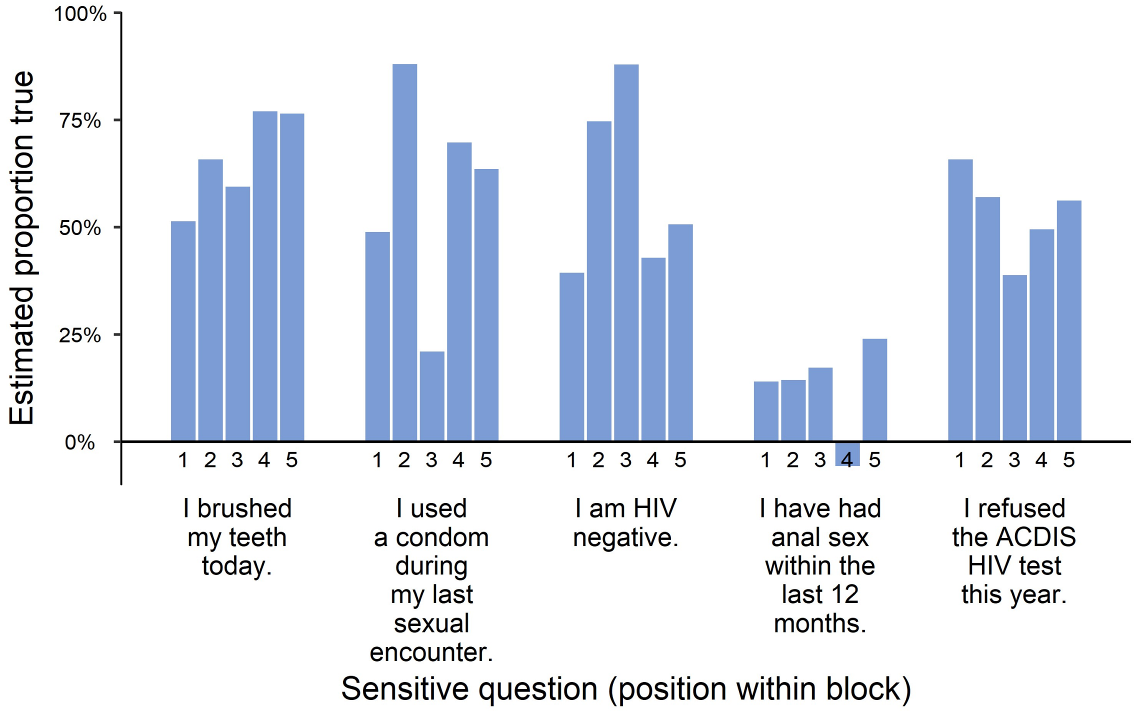

Each bar represented the percentage of sums which are equal to 0, where each sum is the sum of the four non-sensitive question "true" (affirmative) answers associated with a given sensitive question item block.
